# How familiarity warps representation in the face space

**DOI:** 10.1167/jov.20.7.18

**Published:** 2020-07-21

**Authors:** Vassiki Chauhan, Ilona Kotlewska, Sunny Tang, M. Ida Gobbini

**Affiliations:** Department of Psychological and Brain Sciences, Dartmouth College, Hanover, NH, USA; Department of Cognitive Science, Faculty of Humanities, Nicolaus Copernicus University, Torun, Poland; Dipartimento di Medicina Specialistica, Diagnostica e Sperimentale (DIMES), Medical School, University of Bologna, Bologna, Italy; Cognitive Science Program, Dartmouth College, Hanover, NH, USA

**Keywords:** familiarity, learning, face recognition, identity, categorical perception

## Abstract

Recognition of familiar as compared to unfamiliar faces is robust and resistant to marked image distortion or degradation. Here we tested the flexibility of familiar face recognition with a morphing paradigm where the appearance of a personally familiar face was mixed with the appearance of a stranger (Experiment 1) and the appearance of one's own face with the appearance of a familiar face and the appearance of a stranger (Experiment 2). The aim of the two experiments was to assess how categorical boundaries for recognition of identity are affected by familiarity. We found a narrower categorical boundary for the identity of personally familiar faces when they were mixed with unfamiliar identities as compared to the control condition, in which the appearance of two unfamiliar faces was mixed. Our results suggest that familiarity warps the representational geometry of face space, amplifying perceptual distances for small changes in the appearance of familiar faces that are inconsistent with the structural features that define their identities.

## Significance statement

Familiar faces are recognized robustly despite image degradation, differences in lighting, head position, or distance. Here, we investigated the flexibility of familiar face recognition with two separate experiments using a morphing paradigm. Our data suggest that a familiar face occupies a sector of perceptual face space that is expanded relative to its extent based on differences in measured physical similarity. This expansion in representational space may be part of a more general mechanism that could explain how learning can facilitate processing of behaviorally relevant stimuli.

## Introduction

Human beings are adept at detecting, identifying, and discriminating between faces, despite the high degree of visual similarity based on first-order features. A compelling explanation for how we can discriminate different identities reliably comes from the hypothesis that faces are encoded as vectors in a high multidimensional representational space (Valentine, 1991; Lee, Byatt, & Rhodes, 2000; Leopold, O'Toole, Vetter, & Blanz, 2001; Jiang, Blanz, & O'Toole, 2007). Vectors for images of the same identity are located close to each other in this multidimensional face space. Face images for different identities that are located close to each other are harder to discriminate as compared to those that are distant from each other in face space. Several studies suggest that faces we encounter in early life (Slater et al., 2010) or on a regular basis (O'Toole, Deffenbacher, Valentin, & Abdi, 1994) play a dominant role in shaping the dimensions of face space. People can discriminate the identities of faces of their own race better than faces of other races (Feingold, 1914; Rhodes, Tan, Brake, & Taylor, 1989). Webster, Kaping, Mizokami, and Duhamel (2004) showed that if images of faces from one's own race are morphed with images of another race's faces, one tends to perceive morphs of equal mixtures as the other race. The point of subjective equality for mixed-ethnicity morphs is shifted by 8% to 17% toward one's own race.

Prior research shows that faces of personally familiar identities are better discriminated and recognized than unfamiliar faces over changes in head angle, lighting, compression, or squeezing across different exemplars of the same identity (di Oleggio Castello, Taylor, Cavanagh, & Gobbini, 2018; Gilad-Gutnick, Harmatz, Tsourides, Yovel, & Sinha, 2018; Harmon & Julesz, 1973; Hole, George, Eaves, & Rasek, 2002; Sinha, Balas, Ostrovsky, & Russell, 2006). Clearly, the neural face system is capable of efficiently extracting the invariant identity across multiple and varied images of the same familiar individual (Guntupalli & Gobbini, 2017; Guntupalli, Wheeler, & Gobbini, 2017). At the same time, small differences in images of familiar individuals can be discriminated more efficiently for familiar than unfamiliar faces (Chauhan, Visconti di Oleggio Castello, Soltani, & Gobbini, 2017; di Oleggio Castello, Halchenko, Guntupalli, Gors, Gobbini, 2017; di Oleggio Castello, Taylor, Cavanagh, & Gobbini, 2018; di Oleggio Castello, Wheeler, Cipolli, & Gobbini, 2017; Ramon & Gobbini, 2018; Visconti di Oleggio Castello, Guntupalli, Yang, & Gobbini, 2014). The visual appearance of a personally familiar face is learned in detail over protracted and repeated exposure during real-life interactions. In a representational space for faces, the sectors populated by familiar faces may be perceptually expanded because of the rich variety of visual experiences with those faces. Much in the same way that faces of people from one's own race are represented more richly and with more discriminating information, faces of personally familiar others are represented perhaps even more richly.

Here, we asked whether personal familiarity affects categorical changes from one facial identity to another. We created morph continua between pairs of familiar (one's own face or faces of friends) and unfamiliar (faces of strangers) identities and measured how frequently different levels of morphed faces were assigned to the original identities. Morph continua with different levels of mixture of the attributes of two stimuli are used extensively in experimental psychology to test categorical perception. Specifically, in the field of face perception, prior research using face morphs has been focused on studying how flexible categorical perception of faces is in a variety of different conditions such as age, gender, and race (Angeli, Davidoff, & Valentine, 2008; Rhodes, Jeffery, Watson, Clifford, & Nakayama, 2003; Webster, Kaping, Mizokami, & Duhamel, 2004). Categorical perception of a stimulus refers to the idea that for a continuous range of morphs between two stimuli, there is a perception of a categorical change around the midpoint of the morph continuum, and distinctions between morph levels within each category are less distinguishable. This has been shown to be the case for a variety of different stimuli (Harnad, 1987), highlighting how humans tend to perceive continuous variations of stimuli as discrete categories. Categorical perception of facial identity has also been reported for both familiar and unfamiliar stimuli (Beale & Keil, 1995; Kaufmann & Schweinberger, 2004; Kikutani, Roberson, & Hanley, 2008; Ramon & Van Belle, 2016; Rotshtein, Henson, Treves, Driver, & Dolan, 2005). In our experiment, we wanted to assess if a shift in categorical boundary for recognizing an identity is observed when morphing a familiar and unfamiliar identity, reflecting an expansion of perceptual distances for variations among stimuli that are perceived as the familiar individual, in support of the hypothesis that exposure to personally familiar faces alters the representational geometry of face space.

We predicted two possible outcomes. The first hypothesis poses that repeated exposures to personally familiar faces leads to the development of perceptually expanded representational subspaces for familiar individuals. Such an expansion would shift the categorical boundary between familiar and unfamiliar identities, making an equal admixture of attributes of familiar and unfamiliar individuals perceptually resemble the unfamiliar individual. This hypothesis is supported by the work of Stevenage (1998), showing that stricter criteria for naming faces of identical twins develop as a result of training, demonstrating the importance of such criteria for discriminating between very similar faces. Alternatively, under a second hypothesis, multiple exposures to faces of familiar individuals may bias the perception of an ambiguous identity (here, a morphed image) toward being labeled more easily as a familiar individual. This hypothesis is based on the evidence that different image manipulations such as squeezing or compressing images of familiar faces do not seem to disrupt the process of recognition of those identities (Gilad-Gutnick, Harmatz, Tsourides, Yovel, & Sinha, 2018; Sinha, Balas, Ostrovsky, & Russell, 2006), despite the alteration of the shape of the face and the shape of the features. Therefore, the manipulation of the identity information with the use of morphs might result in a stable, enhanced recognition with a larger categorical boundary.

In this article, we present results from two different experiments. In the first study, we used the same set of personally familiar targets and unfamiliar controls for all participants who came from the same social group. In the second study, we tested a set of personally familiar faces that varied across participants and unfamiliar faces and, as a further condition, one's own face.

Results from both experiments show that the midpoint of a morph spectrum between a familiar identity and an unfamiliar identity is more likely to be labeled as the unfamiliar identity, suggesting that we use a more conservative threshold for the process of recognizing faces of familiar identities.

## Experiment 1

### Method

### Participants

Sixteen graduate students from the Dartmouth College community participated in this experiment (five males, 26.8 ± 2.4). Two of the participants were left-handed. For analysis, data for one participant were discarded due to an error in recording responses. Therefore, the results presented in this report are from 15 participants (five males, 26.7 ± 2.4). Sample size was chosen based on the sample sizes used in previous reports investigating categorical perception of facial identity (Jacques & Rossion, 2006; Kaufmann & Schweinberger, 2004; McKone, Jeffery, Boeing, Clifford, & Rhodes, 2014; Natu, Barnett, Hartley, Gomez, Stigliani, & Grill-Spector, 2016). All participants provided written informed consent to participate in the experiment and were compensated with cash for their time. The Dartmouth Committee for the Protection of Human Subjects approved the experiment (Protocol 21200).

### Equipment

Participants sat 50 cm from a computer screen in a dimly lit room. The resolution of the screen was 1,440 × 900 pixels. The experiment was run on a GNU/Linux workstation, and the presentation code was written in MATLAB, using Psychophysics toolbox extensions (Brainard & Vision, 1997; Kleiner, Brainard, & Pelli, 2007).

### Stimuli

**Figure 1.**
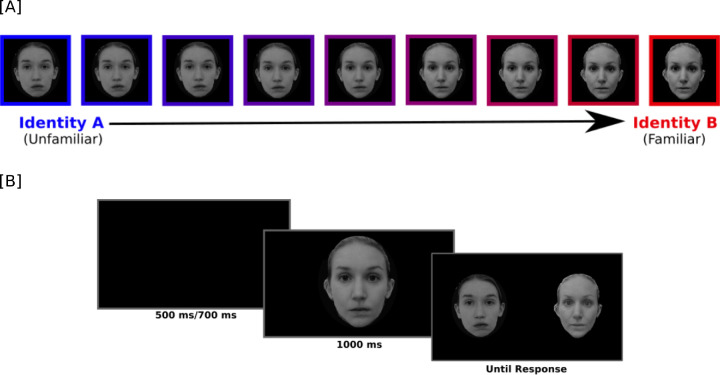
(A) Morph spectrum from an unfamiliar identity to a familiar Identity B. Morphs values ranged from 10% Identity B to 90% Identity B, in steps of 10%. (B) Example sequence from one trial of the experiment.

The stimuli used for this experiment were grayscale pictures of three graduate students that were personally familiar to all participants and three unfamiliar faces that were visually matched with the familiar face identities. A morph continuum between each familiar identity and its visually matched control was created with the software FantaMorph (Abrosoft: https://www.fantamorph.com/). This procedure involved placing around 150 points per image on each of the pairs of face images used to create the morphs. These points lay primarily on the internal features of the face and along the silhouette of the face, as depicted in Figure 1A. The image-processing algorithm implemented in FantaMorph used these points as landmarks to align the two images. By regulating the contribution of each identity for each image, we were able to create morphs of different strengths toward one identity or the other that contributed to the morph, resulting in a morph continuum from one original face image to the other (Figure 1A and Figure 1B). Additionally, with identical procedures as described above, three morph continuums for six independent sets of identities that were all unfamiliar to the participants were created to serve as controls in the experiment. For both experimental and control morph continua, two pairs were male and one pair was female. All of the original pictures for each identity were acquired in a photo studio in the laboratory with the same lighting conditions and the same distance from the camera to minimize low-level visual dissimilarities between stimuli. We also matched the luminance of all stimuli to a target luminance value (128 in RGB) in order to control for differences in visual properties of the images themselves and make the transition from one morph to the other even more homogeneous (Willenbockel et al., 2010).

### Paradigm

Pictures of all the original identities used for the experiment were shown to the participants before starting the experiment. Each face was presented for 4 s, and participants were asked to look at the faces as they would under natural viewing conditions. Each identity was presented once.

In the experiment, each trial sequence started with the presentation of a fixation cross that remained on screen for a jittered interval between 500 and 700 ms. This was followed by the presentation of a target, morphed image for 1,000 ms centered on the location of the fixation cross, subtending 3.5 × 4.5 degrees of visual angle. The target stimuli were morphed images from 10% to 90% in steps of 10%. As soon as the target image disappeared from the screen, the two original identities from which the target morphed image was created were presented on either side of the fixation. The distance between the two original faces was 10 degrees of visual angle. The dimensions were the same for all stimuli. The two test faces stayed on screen until the subjects made a response.

The participants performed a two alternative forced-choice identity recognition task. Participants were asked to respond by pressing the left or right arrow key to indicate which of the two original identities was more similar to the target face (Figure 1B). Participants were instructed to provide their response as quickly as possible, but not at the expense of accuracy.

Three blocks with the familiar/unfamiliar morphs and three blocks with unfamiliar/unfamiliar morphs were presented. Each block consisted of 108 trials, where each morphed identity was repeated four times at each morph percentage (10% to 90%, in steps of 10%). Thus, over the course of the entire experiment, an image from each of the identity continua (Figure 1A) was presented to the participant 12 times. Different images from the same identity continuum were never presented in consecutive trials.

### Data analysis

For the analysis of the percentage of responses, we refer to responses to one of the two faces upon which the morph continuum was built as “Identity B.” In the unfamiliar-familiar morphs, “Identity B” corresponded to the familiar identity. In the control condition with unfamiliar-unfamiliar morphs, the analysis of percentage responses required us to designate one identity per morph continuum as “Identity B,” and this designation was arbitrary. In order to deal with the randomness of this assignment, we decided to fix the responses to 50% for morphs at 50% by flipping the identities of A and B and estimated the variability in “Identity B” responses to different morphs using a bootstrap procedure. Thus, the percentage of “Identity B” responses for 50% morphs was 50% by definition. The percentage of “Identity B” responses for 40% morphs is the average of “Identity B” responses for 40% morphs and 100 minus the percentage of “Identity B” responses for 60% morphs, and so forth. We calculated 95% bootstrapped confidence intervals around these symmetric averages. These are the values that have been reported in the figures and all the tables and the ones we used for the statistical analyses. We analyzed the percentage of Identity B responses by building a generalized linear mixed model with binomial error distribution and logit model as linking function using the R package lme4 (Bates et al., 2015) and the function “Anova” from package “car” (Fox et al., 2012). All figures were made using the library “ggplot2” in R (Wickham, 2011). We constructed the model with the categorical response (“Identity A” or “Identity B”) as the dependent variable, morph percentage and familiarity condition as independent variables, and participant, morph stimulus continuum, and random intercepts for participants as random effects. Scaled values of the morph percentage were used as a continuous variable, whereas the familiarity condition was specified as a categorical variable with a zero-sum contrast. Statistical significance of the main effects and interaction effects was tested using a Type 3 analysis of deviance, as implemented in the package “car” (Fox et al., 2012).

For the analysis of reaction times, we discarded trials that had response times shorter than 150 ms and longer than 5 s. Only correct trials were included in the analysis for reaction time (RT). Trials were considered correct when participants, response choice matched the identity of the face that had a greater contribution to morph target. For example, if the morph was made of 40% Identity A and 60% Identity B, the correct response was considered Identity B. Therefore, this analysis excluded the 50% morph condition, since no “correct” response exists for that condition. A linear mixed model with log-transformed RTs of correct trials as the dependent variable and the morph percentage of the probe and familiarity condition as independent variables was fit to the data. Scaled values of the morph percentage were used a continuous variable, whereas the familiarity condition was specified as a categorical variable with a zero-sum contrast. We also included the participants, morph stimulus continuum, and random intercepts for participants as random effects in the model. The RTs were log transformed in order to fit the assumptions of linear mixed models.

### Data availability

Raw data and the code are available https://github.com/vassiki/CategoricalPerception.

## Results

### Percentage responses

First, we determined the percentage of responses that corresponded to “Identity B” (Figure 1B) of the morph spectrum. For unfamiliar-familiar morphs, “Identity B” indicates the personally familiar identity. The analysis of this dependent variable with a generalized linear mixed model revealed the main effect of morph percentage (χ^2^(1) = 3,596.5, *p* < 0.001) but not of familiarity condition (χ^2^(1) = 0.48, *p* = 0.49). However, the interaction between morph percentage and familiarity condition was significant (χ^2^(1) = 4.9, *p* = 0.03). The mean percentage responses and bootstrapped confidence intervals are included in Tables 1 and 2. Unstandardized effect sizes depicting the difference in familiar and unfamiliar blocks at each morph level on percentage “Identity B” responses are included in Table 3. The effect sizes indicate that the significant interaction between the morph percentage and familiarity condition is driven by the ambiguous, 50% morph between unfamiliar and familiar identities. There is a significant reduction of 8.7% (CI [1.1, 17.6]) in the percentage of “Identity B” (familiar identity) responses in the unfamiliar-familiar morph condition as compared to the unfamiliar-unfamiliar morph condition. There also is a significant effect of familiarity for 40% morphs, which were less likely to be classified as the familiar “Identity B” face in unfamiliar/familiar morphs than as an unfamiliar “Identity B” face in unfamiliar/unfamiliar morphs (16.3% versus 20.2%, Table 1; effect size of 3.9, CI [2.5, 10.0], Table 3).

**Table 1.** Percentage of “Identity B” response for unfamiliar to familiar morph continuum (Identity B corresponds to the familiar identity). Bootstrapped 95% confidence intervals around the percentage responses are reported in the third column.

Morph percentage	Mean percentage response	Bootstrapped 95% CIs
10	5.4	2.8, 8.1
20	6.3	3.1, 10.0
30	7.5	4.8, 10.3
40	16.3	11.7, 21.5
50	41.3	34.6, 47.4
60	79.1	73.1, 83.7
70	91.4	87.6, 94.6
80	95.4	92.4, 98.0
90	97.0	94.1, 98.9

**Table 2.** Percentage of “Identity B” response for unfamiliar to unfamiliar morph continuum. In these blocks, Identity B corresponded to an arbitrary unfamiliar identity. Bootstrapped 95% confidence intervals around the percentage responses are reported in the third column.

Morph percentage	Mean percentage response	Bootstrapped 95% CIs
10	4.8	1.7, 9.4
20	5.9	2.4, 10.2
30	9.2	5.2, 13.9
40	20.2	15.6, 25.1
50	50.0	44.6, 55.2
60	79.8	74.9, 84.4
70	90.8	86.1, 94.8
80	94.1	89.8, 97.6
90	95.2	90.6, 98.3

**Table 3.** Effect sizes for percentage of “Identity B” responses in the unfamiliar-unfamiliar continuum and the unfamiliar-familiar morph continuum across different morph percentages. The values were computed by calculating the difference between percentage “Identity B” responses for the two morph conditions. The third column shows bootstrapped confidence intervals around the difference in percentage responses.

Morph percentage	Effect size	Bootstrapped 95% CIs
10	−0.6	−3.5, 2.8
20	−0.4	−3.2, 2.1
30	1.7	−1.4, 5.0
40	3.9	2.5, 10.0
50	8.7	1.1, 17.6
60	0.7	−5.5, 7.3
70	−0.6	−4.4, 2.7
80	−1.2	−4.5, 1.9
90	−1.8	−4.3, 0.4

**Figure 2.**
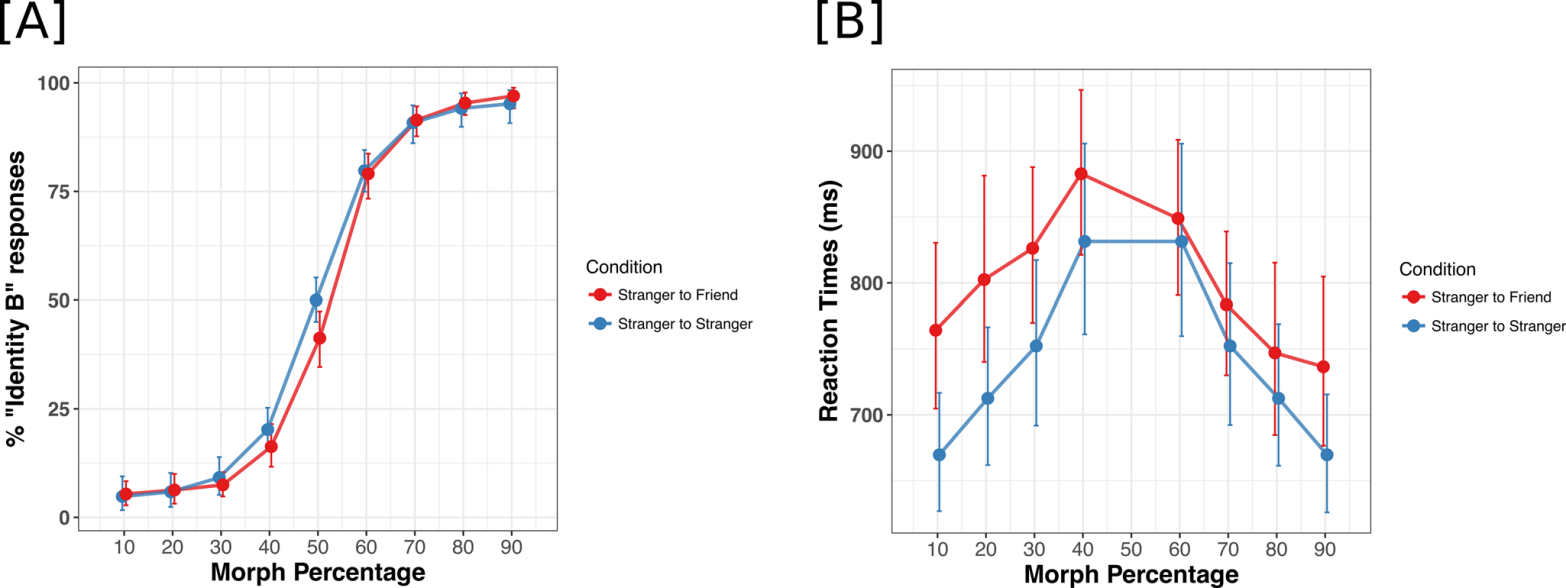
(A) Morph percentage on the x-axis and the mean percentage “Identity B” response on the y-axis. (B) Morph percentage on the x-axis and the mean reaction times (ms) on the y-axis. Morph levels varied from 10% Identity B to 90% Identity B in steps of 10%. In unfamiliar-familiar blocks, “Identity B” corresponds to the familiar face. In unfamiliar-unfamiliar blocks, “Identity B” is arbitrary, and the data points were calculated by bootstrapping across all possible combinations of unfamiliar-unfamiliar morph continua. The error bars represent bootstrapped 95% confidence intervals around the means.

This result indicates that when the identity of morph is ambiguous with some resemblance to a familiar face, people are more conservative and less likely to respond “Identity B” when Identity B corresponds to a familiar face as compared to when both Identities A and B are unfamiliar to participants (Figure 2B).

### Reaction times

The estimates of the model revealed that overall, participants were slower in responding to morphs that contained identity information from familiar exemplars as compared to morphs between two unfamiliar identities (unfamiliar-familiar morph mean RT = 800 ± 51 ms, unfamiliar-unfamiliar morph mean RT = 742 ± 64 ms). Participant's, reaction times were also slower for the more ambiguous identities falling in the middle of the morph continuum. The main effects of familiarity and morph percentage conditions on correct, log-normalized reaction times were significant (familiarity χ^2^(1) = 10.50, *p* = 0.0012 and morph percentage χ^2^(1) = 33.1, *p* < 0.001) (Figure 2A; Tables 4 and 5). The interaction between the two main effects was also found to be significant (χ^2^(1) = 22.01, *p* < 0.001). Estimates of the model determined by using the package lmerTest (Kuznetsova, Brockhoff, & Christensen, 2017) revealed that the interaction between the two main effects was driven by the presence of familiarity information for morphs along the morph continua away from the familiar identities. Means and bootstrapped confidence intervals are included in Tables 4 and 5.

**Table 4.** Average reaction times for correct trials for unfamiliar to familiar morph continuum, across different morphing percentages. Bootstrapped 95% confidence intervals are around mean reaction time.

Morph percentage	Mean reaction time (s)	Bootstrapped 95% CIs
10	0.76	0.70, 0.83
20	0.80	0.74, 0.88
30	0.83	0.77, 0.89
40	0.88	0.82, 0.94
60	0.85	0.79, 0.90
70	0.78	0.73, 0.84
80	0.75	0.69, 0.82
90	0.74	0.68, 0.81

**Table 5.** Average reaction times for correct trials for unfamiliar to unfamiliar morph continuum, across different morphing percentages. Bootstrapped 95% confidence intervals are around mean reaction time.

Morph percentage	Mean reaction time (s)	Bootstrapped 95% CIs
10	0.67	0.63, 0.72
20	0.71	0.66, 0.77
30	0.75	0.69, 0.81
40	0.83	0.76, 0.91
60	0.83	0.76, 0.91
70	0.75	0.69, 0.82
80	0.71	0.66, 0.77
90	0.67	0.63, 0.72

### Interim discussion

In this experiment, we found that participants were more conservative in labeling an ambiguous image as a friend rather than a stranger. We also found that participants were slower in making responses when the morphed image was created using the image of a friend and when morphs were closer to the center of the morph spectrum than the ends. In this experiment, the same images were shown to all participants and the results are based on three identities.

We wanted to further expand our investigation probing a different type of familiarity (with one's own face) and with different identities of personally familiar individuals (friends). Therefore, we ran a second study with a similar task with a more counterbalanced design. By asking participants to bring in their friends for stimulus collection, we ensured that unique faces were used as familiar identities for all participants and that our results are not driven by idiosyncratic visual features of a specific set of personally familiar faces shared across all the participants. Moreover, we included images of one's own face (self) as a special case of facial familiarity. Last, in this second experiment, we intermixed the trial types corresponding to different morph conditions (stranger-friend, stanger-self, friend-self, stranger-stranger, friend-friend) within the same block.

## Experiment 2

### Method

### Participants

Fifteen participants were recruited among the Dartmouth Community (14 undergraduate students and a visiting scholar, *N* = 15) (mean age: 20.1 ± 2.9, all female). All participants were healthy adults with normal or corrected vision. Each participant was accompanied by two of their friends of the same sex to the photo studio in the laboratory at Dartmouth College, where all three individuals were photographed one at a time. All participants provided written informed consent and were compensated with cash for their time. Additionally, all participants signed a model release form in order to allow the use of their photographs as stimuli. The Dartmouth Committee for the Protection of Human Subjects approved the experiments (Protocol 297800).

### Equipment

We used the same equipment as for Experiment 1.

### Stimuli

Five stimulus identities were used for each subject. For each participant, these stimuli included a picture of the participant herself (self), one picture each of two different friends, and one picture each of two different strangers. The images of unfamiliar identities were collected at Massachusetts Institute of Technology, under similar lighting conditions and with the same equipment used to collect the photographs of the participants at Dartmouth College. For each participant, we created five morph spectra using the procedure outlined in Experiment 1, corresponding to the following conditions: stranger with friend, stranger with self, friend with self, stranger with stranger, and friend with friend. Since we used two photographs of friends and two photographs of strangers for each participant, we created one morph continuum for stranger with stranger, one morph continuum for friend with friend, two morph continua for stranger with self, two morph continua for friend with self, and four morph continua for stranger with friend. This procedure resulted in 10 unique morph continua per participant, with nine images per morph continuum (10% to 90% in steps of 10%). Therefore, for each participant, we created 90 stimuli.

### Paradigm

The task for this experiment was identical to Experiment 1. The experimental paradigm was similar to Experiment 1 but, in order to make the task more challenging, we intermixed trials with stimuli from each of the five morph continua. Stimuli were presented in blocks of 90 trials each. The experiment was self-paced, with the participant pressing the spacebar to start each block. Participants performed 10 blocks, with an overall presentation of 10 times for each unique stimulus.

### Data analysis

We analyzed participant's, responses designating one face of each morph continuum as “Identity B.” For morphs between the face of a friend and the face of a stranger, the “more familiar,” or “Identity B,” category corresponded to the face of a friend, and we calculated the percentage of times the participants reported that the morph resembled their friend's face. For morphs between one's own face and the face of a friend, we defined one's own face as the “more familiar,” or “Identity B,” category and calculated the percentage of times the participants reported that a morph between their own face and the face of a friend resembled their own face. For morphs between two strangers and morphs between two friends, we made the percentage responses for these two morph conditions symmetric around the 50% morph. We flipped labels for which identity was designated as “Identity B,” and collapsed the responses across the flipped labels. Reaction times were analyzed similarly to Experiment 1.

### Data availability

As for Experiment 1, raw data and the code are available https://github.com/vassiki/CategoricalPerception.

## Results

### Percentage responses

**Figure 3.**
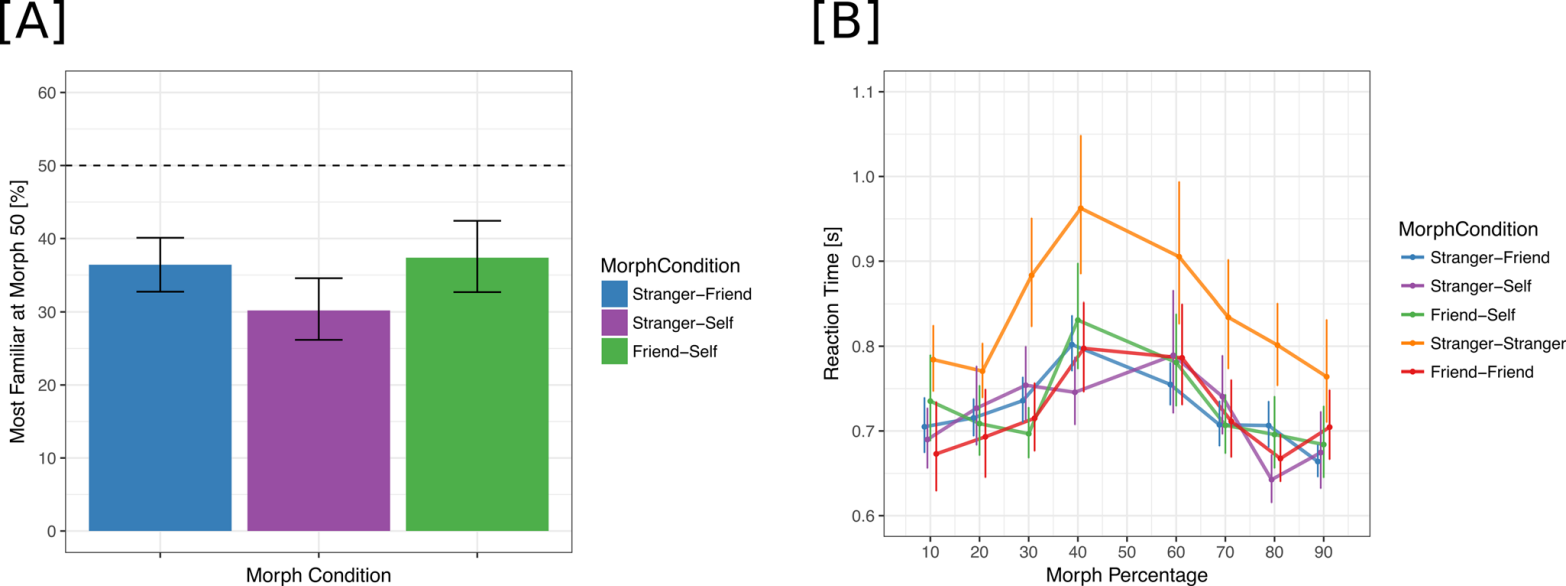
(A) Percentage of “Identity B” responses for a 50% morph between two identities. The more familiar “Identity B” identities were the “Friend” for stranger-friend morphs, “Self” for stranger-self morphs, and “Self” for friend-self morphs. (B) Reaction times for correct trials as a function of morph percentage; colors represent morph condition.

**Figure 4.**
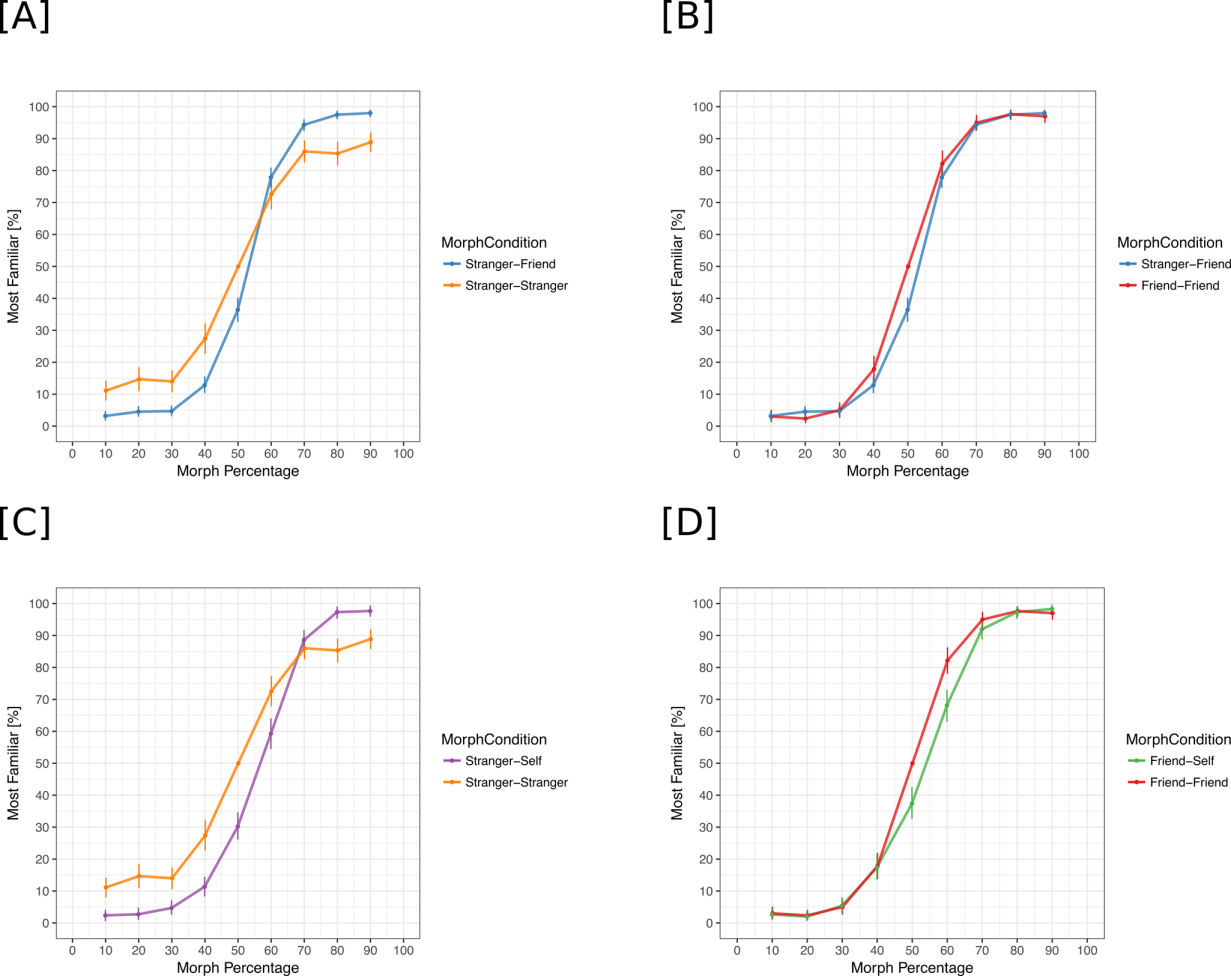
Percentage “Identity B” responses as a function of morph percentage. (A) Percentage “Identity B” responses for stranger-stranger and stranger-friend morphs. (B) Percentage“Identity B” responses for friend-friend and stranger-friend morphs. (C) Percentage “Identity B” responses for stranger-stranger and stranger-self morphs. (D) Percentage “Identity B” responses for friend-friend and friend-self morphs.

The analysis of percentage “Identity B” responses revealed a significant main effect of scaled morph percentage (χ^2^(1) = 2,736.8, *p* < 0.001) but not of morph condition (χ^2^(4) = 6.00, *p* = 0.19). Similar to Experiment 1, we found a significant interaction between scaled morph percentage and morph condition (χ^2^(4) = 243.93, *p* < 0.001) (Figure 4; Table 6). The 50% morph for the stranger with friend condition was labeled as “Friend” 36% of the time [33, 40], similar to the results in Experiment 1. The 50% morph for the stranger with self condition was labeled as “Self” 30% of the time [26, 35], and the 50% morph for the friend with self condition was labeled as “Self” 37% of the time [33, 40] (Figure 3A). The effect sizes for all morph percentages are included in Tables 7, 8, and 9.

**Table 6.** Percentage “more familiar” or “Identity B” responses for each morph condition and morph percentage. Bootstrapped 95% confidence intervals are calculated around mean accuracy in each condition.

Morph condition	Morph percentage	Percentage “most familiar” responses	Bootstrapped 95% CIs
Stranger with friend	10	3.2	1.8, 4.5
	20	4.5	3.0, 6.2
	30	4.7	3.2, 6.4
	40	12.8	10.3, 15.3
	50	36.4	32.7, 39.3
	60	77.9	74.8, 81.0
	70	94.3	92.5, 96.2
	80	97.5	96.2, 98.7
	90	98.0	96.8, 99.0
Stranger with self	10	2.4	0.7, 4.4
	20	2.7	1.0, 4.8
	30	4.7	2.7, 7.1
	40	11.4	8.4, 14.4
	50	30.2	26.2, 34.6
	60	59.3	54.6, 64.0
	70	86.0	82.3, 89.3
	80	85.3	81.7, 89.0
	90	88.9	85.9, 91.9
Friend with self	10	2.7	1.0, 4.3
	20	2.0	0.7, 3.7
	30	5.4	3.0, 8.0
	40	17.4	13.7, 21.4
	50	37.4	32.6, 42.4
	60	68.1	63.1, 72.8
	70	91.9	88.9, 94.9
	80	97.3	95.3, 98.9
	90	98.3	96.9, 99.7
Stranger with stranger	10	11.1	8.1, 14.1
	20	14.7	11.0, 18.3
	30	14.0	10.7, 17.7
	40	27.4	22.7, 32.1
	50	50.0	50, 50
	60	72.6	67.9, 77.3
	70	86.0	82.3, 89.3
	80	85.3	81.7, 89.0
	90	88.9	85.9, 91.9
Friend with friend	10	3.0	1.3, 5.0
	20	2.4	1.0, 4.1
	30	5.0	2.7, 7.4
	40	17.9	13.8, 22.0
	50	50.0	50, 50
	60	82.2	78.1, 86.2
	70	95.0	92.6, 97.3
	80	97.6	95.9, 99.0
	90	97.0	95.0, 98.6

**Table 7.** Effect sizes for percentage more familiar “Identity B” responses in the stranger-friend morph condition. These values were computed by comparing the percentage more familiar response at each morph percentage with the percentage response for the same morph percentage in the stranger-stranger morph condition. Negative values indicate that the stranger was chosen as a label more frequently than the friend.

Morph percentage	Effect size	Bootstrapped 95% CIs
10	−7.9	−11.3, −4.6
20	−10.1	−14.3, −6.1
30	−9.3	−13.2, −5.5
40	−14.6	−19.8, −9.3
50	−13.6	−17.1, −9.9
60	5.3	−0.3, 10.8
70	8.3	4.5, 12.3
80	12.6	8.3, 16.2
90	9.1	5.9, 12.3

**Table 8.** Effect sizes for percentage more familiar responses in the stranger-self morph condition. These values were computed by comparing the percentage more familiar response at each morph percentage with the percentage response for the same morph percentage in the stranger-stranger morph condition. Negative values indicate that the stranger was chosen as a label more frequently than the friend.

Morph percentage	Effect size	Bootstrapped 95% CIs
10	−8.7	−12.12, −5.4
20	−12.0	−16.0, −7.9
30	−9.3	−13.3, −5.0
40	−16.1	−21.7, −10.7
50	−19.8	−23.8, −15.4
60	−13.3	−20.0, −6.6
70	2.6	−2.1, 7.3
80	12.0	8.0, 16.0
90	8.7	5.4, 12.1

**Table 9.** Effect sizes for percentage more familiar responses in the friend-self morph condition. These values were computed by comparing the percentage more familiar response at each morph percentage with the percentage response for the same morph percentage in the friend-friend morph condition. Negative values indicate that the friend was chosen as a label more frequently than the self.

Morph percentage	Effect size	Bootstrapped 95% CIs
10	−0.3	−3.0, 2.0
20	−0.4	−2.7, 2.0
30	0.3	−3.0, 3.7
40	−0.5	−6.2, 5.3
50	−12.6	−17.3, −7.6
60	−14.0	−20.4, −8.0
70	−3.1	−7.1, 0.6
80	−0.3	−2.7, 2.0
90	1.3	−1.0, 3.8

In order to quantify the preference for the “more familiar” identity across morph conditions, we computed the asymmetry bias in the responses using the following equation:

*bias*(*x*, *y*) = *f*(*x*) − (100 − *f*(*y*)), where *x* ∈ {10, 20, 30, 40, 50}; *y* = 100 − *x*.

**Figure 5.**
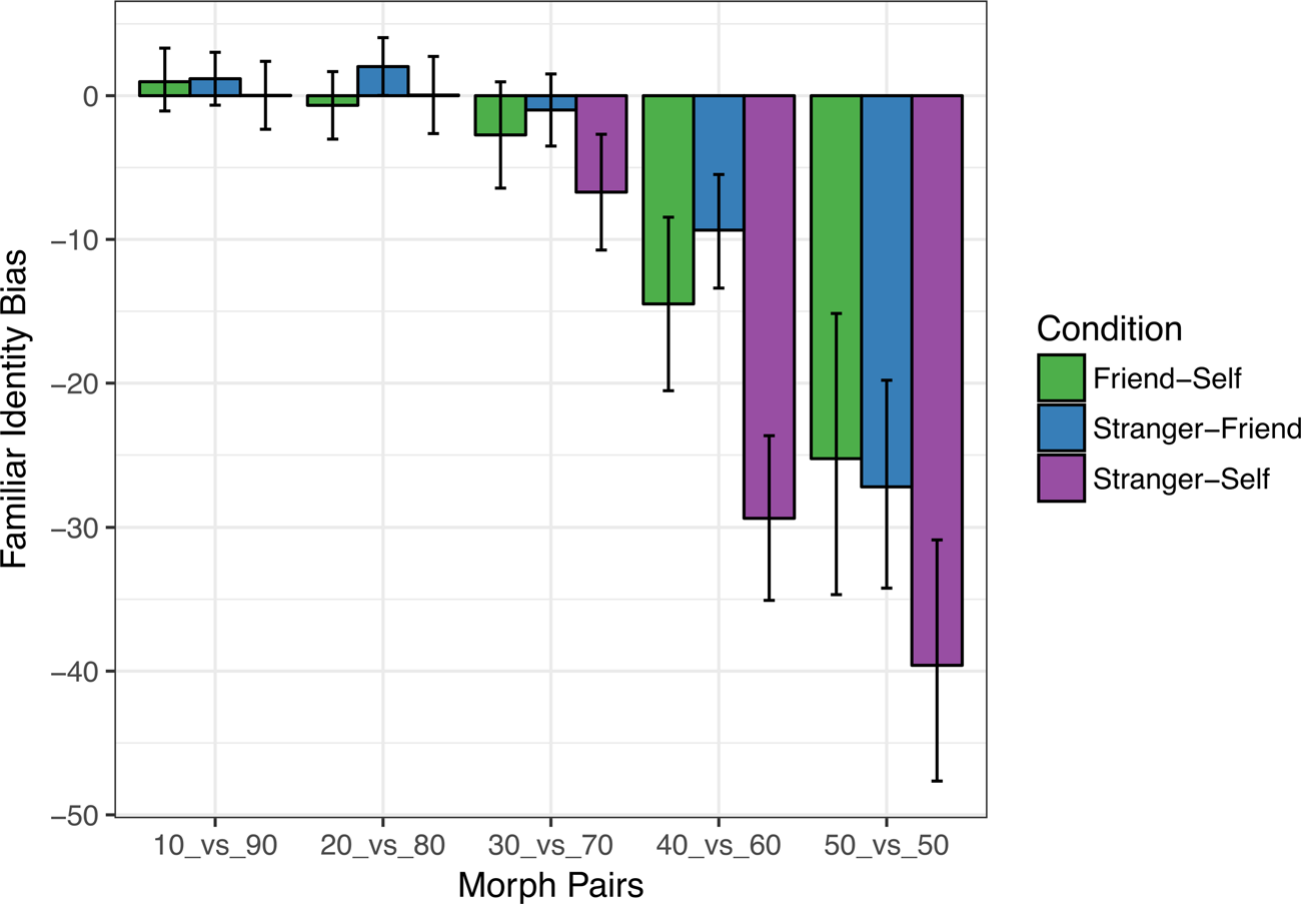
Asymmetry bias for pairs of morph percentages distributed symmetrically around 50%. Across all morph conditions, larger negative biases indicate a more conservative threshold for labeling a morphed image as the more familiar identity.

*f*(*x*) is the percentage “most familiar” response to morph percentage *x*. For a morph percentage of 50, *f*(*y*) was set to the value of 50. Negative values of bias indicate a preference for the less familiar identity within a given morph condition (Figure 5). For morph percentages close to 50% and 40% versus 60%, we observe significantly negative asymmetry biases, suggesting that participants are more conservative in using the “more familiar” label as the morph identity becomes more ambiguous.

### Reaction times

Reaction times were found to be slower for stranger with stranger morphs as compared to all other conditions (Figure 3B, Table 10). Moreover, we found slower reaction times for morphs in the middle (e.g., 40%–60%) as compared to the ends of the morph spectrum (90-80%, 10-20%) (Figure 3B, Table 10). Analysis of log-transformed reaction times in correct trials revealed a main effect of morph condition (χ^2^(4) = 18.39, *p* = 0.001) and scaled morph percentage (χ^2^(1) = 14.62, *p* < 0.001). The interaction between morph condition and scaled morph percentage was not found to be significant (χ^2^(4) = 9.16, *p* = 0.06). In Experiment 2, the direction of results of the reaction time is in contrast with those from Experiment 1. This discrepancy could be due to the decision of presenting the trials from all conditions intermixed within blocks, unlike the design of Experiment 1, where the trials of one condition (e.g., morph continua between a familiar and an unfamiliar identity) were presented within the same block. Presenting trials of different conditions intermixed in the same block might have forced the participants to use the same response strategy for all morph conditions, as opposed to performing the task as they did in Experiment 1, where the identity for the response was expected in advance.

**Table 10.** Average reaction times for each morph condition and morph percentage. Bootstrapped 95% confidence intervals are calculated around mean reaction time.

Morph condition	Morph percentage	Mean reaction time (s)	Bootstrapped 95% CIs
Stranger with friend	10	0.70	0.67, 0.74
	20	0.72	0.69, 0.74
	30	0.74	0.71, 0.76
	40	0.80	0.77, 0.84
	60	0.75	0.73, 0.78
	70	0.71	0.68, 0.74
	80	0.71	0.68, 0.74
	90	0.66	0.65, 0.68
Stranger with self	10	0.69	0.66, 0.73
	20	0.73	0.68, 0.78
	30	0.75	0.71, 0.80
	40	0.75	0.71, 0.79
	60	0.79	0.72, 0.87
	70	0.74	0.70, 0.79
	80	0.64	0.62, 0.67
	90	0.67	0.63, 0.72
Friend with self	10	0.74	0.69, 0.79
	20	0.71	0.67, 0.75
	30	0.70	0.67, 0.73
	40	0.83	0.77, 0.90
	60	0.78	0.73, 0.84
	70	0.71	0.67, 0.74
	80	0.70	0.66, 0.74
	90	0.68	0.64, 0.73
Stranger with stranger	10	0.78	0.75, 0.82
	20	0.77	0.74, 0.80
	30	0.88	0.82, 0.95
	40	0.96	0.88, 1.05
	60	0.91	0.83, 0.99
	70	0.83	0.77, 0.90
	80	0.80	0.75, 0.85
	90	0.76	0.71, 0.83
Friend with friend	10	0.67	0.63, 0.73
	20	0.69	0.64, 0.75
	30	0.72	0.68, 0.76
	40	0.80	0.75, 0.85
	60	0.79	0.73, 0.85
	70	0.71	0.67, 0.76
	80	0.67	0.64, 0.70
	90	0.70	0.67, 0.75

### Interim discussion

In this experiment, we replicated the main finding from Experiment 1, demonstrating that participants were less likely to label an ambiguous face as the more familiar identity, rather than the less familiar or unfamiliar identity. The size of this effect was remarkably consistent across three different familiarity contrasts, ranging from 12.6% to 19.8% for 50% morphs, and larger than the effect size in Experiment 1 of 8.7%. We also found that the participants were less accurate and slower in performing the task for the Stranger with Stranger morph spectrum, in contrast to Experiment 1, possibly due to the use of intermixed trial types in this experiment.

## Discussion

We investigated how categorical boundaries of identity are influenced by familiarity. We tested categorical decisions about recognition of identities using morphs between different identities. Using this experimental design, previous work has shown that perception of facial identity is “categorical,” reflected in the abrupt transitions in perception of a different identity somewhere along the morph continuum (Beale & Keil, 1995; Ramon & Van Belle, 2016; Rotshtein, Henson, Treves, Driver, & Dolan, 2005). Here, in our first experiment (Experiment 1), unlike previous work reported in the literature, we tested morph continua that were created with a familiar and an unfamiliar identity. Results showed that the categorical decision boundary was shifted toward the personally familiar faces, such that the morphed image at the midpoint was more often judged to be the unfamiliar individual. In our second experiment (Experiment 2), we replicated this result and showed further that the categorical boundary is shifted similarly for one's own face when morphed with the face of strangers or the face of personally familiar others. This finding further supports our hypothesis that the higher degree of familiarity with the appearance of a face affects the categorical boundary that distinguishes that identity from other identities. While familiar faces are flexibly recognized in highly degraded or distorted images (Gilad-Gutnick, Harmatz, Tsourides, Yovel, & Sinha, 2018; Sinha, Balas, Ostrovsky, & Russell, 2006), a more conservative approach is used in labeling an ambiguous identity as a familiar individual when noise from an unfamiliar identity is added to the features of the familiar face. In the light of this result, we propose that multiple exposures to the same individual in a variety of different viewing conditions sharpen tuning to the features that make that familiar identity distinct (Tanaka, Giles, Kremen, & Simon, 1998). Conversely, an ambiguous identity is more likely to be classified as a stranger despite some resemblance to a familiar face.

Previous research has provided evidence for categorical discrimination between identities at around the midpoint of the morph continua when two unfamiliar identities are morphed together (Beale & Keil, 1995). When presented with two alternatives for choosing the identity of a morphed face, subjects are able to choose the correct identity with high accuracy if they are familiar with the original identities (Ramon & Van Belle, 2016). In our experiments, the perception of categorical change of identity was closer to the end represented by the familiar identity, indicating that when presented with an ambiguous identity, the visual system is less sensitive to changes in the features of an unfamiliar face. Our results provide strong evidence that learning through a repeated and prolonged exposure to familiar faces warps the representational geometry of face space, resulting in more conservative boundaries for recognition of those familiar identities. Previous research by Wilson and Diaconescu (2006) supports this interpretation by demonstrating that the discriminability between schematic faces that have been learned and visually matched controls improves as a result of training.

Our hypothesis is that multiple exposures to faces of familiar individuals under a variety of viewing conditions such as different head views, facial expressions, differences in lighting, and so on result in flexible, enriched representations of these identities that are resilient to distortions in visual features. These enriched representations afford robust recognition across diverse conditions, even when images of familiar individuals are experimentally manipulated by squeezing, flattening, or caricaturization (Gilad-Gutnick, Harmatz, Tsourides, Yovel, & Sinha, 2018; Sinha, Balas, Ostrovsky, & Russell, 2006). At the same time, learning these representations increases our sensitivity to features that are inconsistent with them. For example, distinctions between faces of two siblings of the same sex, or even identical twins, are more easy to discern to family members than they are to strangers (Stevenage, 1998).

Our results are in line with research on face perception of other races. A study by Webster and colleagues (2004) showed that perception of race is influenced by the set of faces that observers are exposed to and that participants have a directional bias in determining the categorical boundary for morphs between two races (Japanese and Caucasian). The direction of this bias is determined by the participants' race. The categorical boundary for a racially ambiguous face was closer to the Japanese face end of the morph spectrum for Japanese observers as compared to Caucasian observers and vice versa. This result suggests that participants use a narrower boundary for categorizing exemplars from their own race due to greater exposure to the features of faces of that race. Similar to these results, our study shows that the categorical boundary for perceiving a face as a familiar individual is shifted toward the familiar original identity.

Our interpretation that personal familiarity changes the geometry of representational space warrants an explanation under the multidimensional face space hypothesis (Valentine, 1991). The norm-based face space hypothesis posits that the average of all the faces encountered by an individual represents the center of a multidimensional space, and unique faces are encoded as points within that space. Tanaka, Giles, Kremen, and Simon (1998) proposed that a face that is atypical, or further away from the norm of the face space, will dominate a 50-50 morph with a more typical face. The authors propose that atypical faces are easier to discriminate than typical faces. This is because representations of atypical faces have larger representational spaces in the absence of a high density of face exemplars competing to occupy the same sectors of the face space. This suggests the possibility that a familiar face similarly occupies an expanded representational space, even though its sector of face space is close to the norm determined by experience and, therefore, should have a high density of face exemplars to compete with. Thus, face space appears to be warped to allocate increased representational space to familiar faces and facilitate discrimination from other face exemplars. Faerber, Kaufmann, Leder, Martin, and Schweinberger (2016) showed that faces of familiar international celebrities are rated as being less typical than their corresponding antifaces, which is not true for faces of strangers (Austrian celebrities and their corresponding antifaces), even though a face and its antiface are equidistant from the norm. Moreover, both may be relatively close to the norm—celebrities often have very regular features—and, therefore, are competing with a high density of other face exemplars. The original face of the familiar celebrity is a distinct point in the multidimensional space, and its antiface is the point that is equidistant from the average in the same space but in the opposite direction. If the subspace for familiar faces in face space is expanded, the perceptual distance between the familiar face and the population average is larger than the perceptual distance between the unfamiliar antiface and the population average, even though these two distances are equal in terms of physical differences. This finding is in line with our results.

In conclusion, our experiment shows that personal familiarity warps face space. A familiar face appears to occupy a sector of perceptual face space that is expanded relative to its size based on differences in measured physical similarity. This expansion of face space for familiar others can enhance view-invariant perception of the identity of a personally familiar other (Jenkins, White, Van Montfort, & Burton, 2011) as well as perception of changes in appearance that have social significance (Chauhan, Visconti di Oleggio Castello, Soltani, & Gobbini, 2017; Visconti di Oleggio Castello, Guntupalli, Yang, & Gobbini, 2014). The expanded representational space for a familiar face allows recognition of identity across varied distortions but at the same time increases the signal to noise to detect features that are inconsistent with an identity.
